# Prevalence of and Risk Factors for Back Pain Among Young Male Conscripts During Compulsory Finnish Military Service

**DOI:** 10.1093/milmed/usab375

**Published:** 2022-01-25

**Authors:** Saara Suikkanen, Harri Pihlajamäki, Mickael Parviainen, Hannu Kautiainen, Ilkka Kiviranta

**Affiliations:** Department of Orthopaedics and Traumatology, Clinicum, University of Helsinki, Helsinki 00014, Finland; Department of Orthopaedics and Traumatology, Seinäjoki Central Hospital, Seinäjoki 60220, Finland; Faculty of Medicine and Life Sciences, University of Tampere, Tampere 33100, Finland; Mehiläinen Medical Centre, Helsinki 00260, Finland; Unit of Primary Health Care, University of Helsinki and Helsinki University Hospital, Helsinki 00029, Finland; Department of General Practice, University of Helsinki, Helsinki 00014, Finland; Department of Orthopaedics and Traumatology, Clinicum, University of Helsinki, Helsinki 00014, Finland; Department of Orthopaedics and Traumatology, Helsinki University Hospital, Helsinki 00260, Finland

## Abstract

**Introduction:**

Back pain is a major reason for sick leaves and disability pension in primary health care. The prevalence of back pain among adolescents and young adults is believed to be increasing, and back pain during military service predicts unspecified back pain during later life. The aim of this study was to investigate the prevalence and risk factors of back pain among conscripts in compulsory Finnish military service during the period 1987-2005.

**Materials and Methods:**

The Finnish Defence Forces recruit all men aged 18 years for compulsory military service, and new conscripts enter the service twice a year. Before entering the service, all conscripts must pass a medical examination and conscripts entering the service are generally healthy.

Health care in Finnish military service is organized by the public Garrison Health Center, and all medical records are stored as part of the Finnish health care operation plan. For this study, we randomly selected 5,000 men from the Finnish Population Register Centre, according to their year of birth from five different age categories (1969, 1974, 1979, 1984, and 1989).

**Results:**

We gathered 4,029 documents for the analysis. The incidence of back pain varied between 18% and 21% and remained unchanged during the examination period. The risk factors for back pain were smoking (risk ratio 1.35, *P*-value <.001), elementary school only as education (risk ratio 1.55, *P*-value <.001), and back problems reported before military service (risk ratio 2.03, *P*-value .002). Half of the back pain incidences occurred during the first months of service.

**Conclusions:**

The prevalence of back pain among male Finnish military service conscripts has not changed in the last 25 years. Twenty percent of conscripts suffer from back-related problems during their military service. The majority of the visits to health centers occurred in the first service months. The risk factors for back pain include smoking, low education level, and musculoskeletal disorders in general. Educating the young people about harms of tobacco and supporting education is a way to influence the back pain prevalence. Strength of this study is a good generalized population sample of young Finnish adult males because of the fact that the Finnish military service is compulsory for all men. All medical records of all visits to the Garrison Health Care Centre were available, and all the conscripts filled the same pre-service questionnaire, minimizing the possibility of selection bias. The sample size was also large. Weakness of this study is that the service time changed during the study period and in the latest conscript group born in 1989, data collection and the data available for this cohort was limited, because nearly half of the conscripts had not yet started their service. The Finnish military service is compulsory only for men and because of the low number of female conscripts, they were excluded from this study. Diagnoses were also missing from 70% of the back-related visits, and these visits were recorded as back pain-related visits according to the reason for seeking care.

## INTRODUCTION

Back pain is one of the main reasons for visiting a physician and a major cause of sick leaves.^1^ The financial burden to society and the humane suffering caused by back pain-related problems is considerable, and prevention methods are of high importance.^2^ The prevalence of back pain is high among the older population and also among adolescents and young adults.^2,3^ Finnish military service conscripts are no different in this respect, and back pain during military service predicts unspecified back pain during later life.^2,3^

The Finnish Defence Forces recruit all men aged 18 years for compulsory military service. One can be exempted for health or religious reasons or can alternatively choose civilian service. New conscripts enter the military service twice a year, in January and in July. Before entering the service, all conscripts must pass a medical examination. Conscripts suffering from diseases that could jeopardize their service are exempted. Conscripts are generally healthy and do not suffer from difficult back problems when military service begins.

The service begins with an 8-week basic training period containing 24 h/week of physical activity of increasing intensity.^4^ At the end of the basic training period, the conscripts gain the fitness level required for military training. After the 8-week basic training period, the physical exercise level is reduced to 15 h/week, but the intensity of the military training increases over the next 4 months. The length of service depends on the assigned position and training. Until 1995, the military service lasted 9 or 11 months, but since then, service periods have been 6, 9, or 12 months. Women have been able to apply for voluntary military service since 1994. The number of female conscripts is less than 3%.

Nearly 80% of all Finnish men start military service, and about 15% of conscripts discontinue service every year.^5^ Musculoskeletal disorder and mental problems are the main reasons for discontinuing, and musculoskeletal disorders show an increasing trend.^6^ The majority of musculoskeletal problems during military service are related to back pain and lower extremity problems.^6^

Little is known about back pain prevalence among conscripts during military service. Most research has been conducted in recruiter forces ^7^ or has focused on hospitalization caused by back pain.^6^ Because nearly all Finnish men participate in military service, the Finnish Defence Forces provide a good data sample for population-based studies.

The aim of this investigation was to study the risk factors for back pain and its prevalence among Finnish male conscripts.

**TABLE I.** Number of Conscripts Visiting the Garrison Health Care Centre because of Back Pain on Different Age Categories

Year of birth	1969	1974	1979	1984	1989
Back-related visits to physician during military service, *n* (%)^a^	166 (21%)	165 (18%)	179 (20%)	167 (19%)	104 (20%)
No back-related visits to physician during military service, *n* (%)	642 (79%)	752 (82%)	720 (80%)	712 (81%)	422 (80%)

a *P*-value for difference between different service years was .71. The proportion of conscripts suffering back pain has remained stable during the study period.

## MATERIALS AND METHODS

We randomly selected 5,000 men from the Finnish Population Register Centre according to their year of birth (1969, 1974, 1979, 1984, and 1989). Each age cohort comprised 1,000 men.

Of the participants selected for this study, 971 were discharged from the data because they did not complete their service during the examination period because of health reasons or they carried out civil service or postponed their service. Finally, we collected the data of 4,029 army conscripts for analysis.

When entering military service, all conscripts complete a pre-service questionnaire assessing their socio-economical and general health factors. This questionnaire is described.^21^

We carefully assessed all visits to the Garrison Health Care Centre during military service from military service documents. Visits to the Garrison Health Care Centre resulting in a back-related diagnosis or when a back problem was the reason for seeking care were recorded as a back-related visit.

## STATISTICAL ANALYSES

The data are presented as means with SD or 95% CIs. The 95% confidence interval was calculated using a bootstrap with 1,000 repetitions. We tested the statistical significance of means between two groups using the *t*-test, and the means between multiple groups using one-way analysis of variance. The Kaplan-Mayer estimate was used for time-to-event analysis. A multivariate Poisson regression model with a robust estimate of variance was used to calculate incidence risk ratios. We tested the significance of the factors using a calculated *z*-value of standardized normal distribution. The STATA 13.1, StataCorp LP (College Station, TX, USA) statistical package was used for all statistical analyses.

The Medical Ethics Committee of the Finnish Defence Forces approved the study plan. The Ethics Committee of the Hospital District of Helsinki and Uusimaa also approved the study (designated 267/13/03/09).

## RESULTS

The mean age of the men at the beginning of military service was 19.2 years (SD 1.1), and their body mass index (BMI) 23.3 kg/m2 (SD 3.8 Kg/m2).

Altogether 781 of the 4,029 study participants (19.4%) visited the Garrison Health Centre because of back-related problems. The number of men with back problems has remained stable over the years (Table I). The incidence was 268 visits per 1,000 conscript years (95% CI: 250-287).

The total number of visits to the Garrison Health Care Centre because of back pain was 1,694. About 80% of the conscripts did not complain of back-related problems during their service, and less than 10% of the conscripts visited the Garrison Health Care Centre more than once because of back-related problems. Of the 4,029 participating conscripts, 191 (5%) visited the Garrison Heath Centre more than twice because of back pain. This 5% of the conscripts contributed to 55% of back pain-related visits to the Garrison Health Centre.

Seventy percent of the back-related visits to the Garrison Health Care Centre resulted in no diagnosis. These visits were recorded as back-related visits, according to the reason for care seeking. Thirty percent of visits resulted in a back-related diagnosis, 95% of which were unspecified back pain. One percent of the diagnoses were scoliosis-related back pain and two percent sciatic back pain. The remaining 3% were classified as having osteoarthritis of the spine or other connective tissue disorders.

**FIGURE 1.**
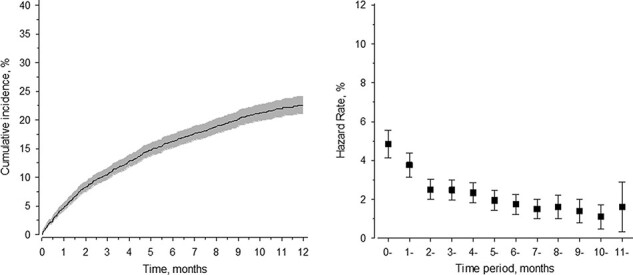
Cumulative incidence and hazard rate of back pain during military service. Half of the visits to Garrison Health occurred during the first 2 months of service. The hazard rate of visiting the Garrison Heath Centre because of back problems was highest during the first and second service months.

Half of the visits occurred during the first 2 months of training (Fig. 1). The hazard rate of visiting the Garrison Heath Centre because of back problems was highest during the first and second service months, after which it gradually decreased (Fig. 1).

Smoking and low education level before service and back problems reported before military service were associated with back pain during military service predicting visits to the Garrison Health Care Centre. Body mass index had no influence on the incidence of visits (Table II).

**TABLE II.** Incident Risk Ratio of Back Related Visits to Garrison Health Care. The General Infroamtion, except BMI, are Gathered from a Pre-service Questionnaire Filled by All Conscritps Prior the Military Service

General information	Incident risk ratio (95% CI)	*P*-value (*P*>|*z*|, two-sided test)
BMI^a^	1.00 (0.99 to 1.03)	.41
Elementary school education only	1.55 (1.30 to 1.84)	<.001
Exercise over two times/week	1.10 (0.93 to 1.29)	.27
Smoking	1.35 (1.14 to 1.60)	<.001
Self-reported previous accident	1.00 (0.79 to 1.26)	.99
Musculoskeletal symptoms reported before military service	0.97 (0.66 to 1.43)	.88
Respiratory symptoms reported before military service	1.04 (0.86 to 1.25)	.69
Gastrointestinal symptoms reported before military service	1.14 (0.50 to 1.18)	.59
Psychological disorders reported before service	0.77 (0.50 to 1.18)	.23
Back problems reported before military service	2.03 (1.29 to 3.19)	.002

Abbreviation: BMI; Body mass index.^a^BMI during the military service. Smoking, low education level, and back pain before the military service were associated with back pain suffered during military service.

93% of the conscripts had BMI 30 or less, and 76% of the conscripts were considered normal or underweight (BMI 25 or less). Visits to Garrison Heath Care because of low back pain did not differ between different BMI categories (Table III).

**TABLE III.** Back Pain-Related Visits to Garrison Health Care according to BMI Category

BMI category	BMI <18 (underweight)	BMI 18-25 (normal weight)	BMI 25-30 (overweight)	BMI >30 (obesity and severe obesity)
No back-related visits to Garrison Health Care	81% ^a^(89^b^)	81% (2,399)	78% (551)	79% (209)
One back-related visit to Garrison Health Care	9% (10^b^)	11% (316)	12% (85)	8% (20)
2-3 back-related visits to Garrison Health Care	8% (9)	5% (151)	7% (49)	9% (24)
> 3 back-related visits to Garrison Health Care	2% (2)	3% (85)	3% (19)	4% (11)

Abbreviation: BMI; Body mass index.^a^Percent of conscripts in the BMI group.

b Absolute number of conscripts analysis of variance one-way test between groups was 0.13. The proportion of obese (BMI over 30) conscripts was 7%.

## DISCUSSION

This study showed that the number of conscripts suffering from back problems during military service has not changed during the 20-year time-period studied. Conscripts are most vulnerable to back problems during the first 2 months of service, after which the risk gradually decreases. Reported low education level, smoking, and musculoskeletal disorders before entering service, including back-related problems, increase the risk of developing back problems during service.

Approximately, 20% of Finnish military service conscripts visit the Garrison Health Centre because of back-related problems. A previous study of 15-64 year-old Finnish citizens showed similar results concerning the prevalence of diagnosed back problems.^8^ A study focusing on musculoskeletal disorders among Finnish conscripts during military service in 1967-2006 showed a 1.6-fold increase of health service utilization, mainly for lower limb and back problems^.6^ According to these findings the increase of health service utilization among Finnish conscripts because of back problems is not because of an increased amount of patients, but rather because of an increased number of visits to a physician per one patient.

The incidence of back pain during military service was 268 visits per 1,000 conscript years. This is clearly higher than that in the U.S. Military Service of active duty members, which is 40.5 per 1,000 conscript years.^7^ This can be explained by the difference of the military servants in these armies. In Finland, the military service is compulsory for all men, whereas in the USA, the military service is recruitment based.

Most of the conscripts who visit the Garrison Health Care Centre visit the physician only once or twice during their military service because of back pain, and most of these visits take place during the first months of service. The physical condition of conscripts is diverse when they enter military service. Poor physical fitness increases the risk of back pain during military service.^9^ On the other hand, heavy physical load also increases the risk of back pain^10^ and the amount of physical activity at the beginning of the Finnish military service for those with a sedentary background can be considered heavy (24 h/week of physical training with increasing intensity). The discrepancy between poor physical condition and physically heavy training is most likely one reason for back-related problems occurring during the first months of service.

Low education level increased the risk for back pain 1.35-fold in this study. Low education level is a known risk factor for back pain.^10,11^ The mechanism of low education level causing back pain is unknown. One explanation is that people with a lower education level tend to work in more physically demanding jobs as they have less work options.^12^ However, occupation explains only part of the phenomena.^12^ In Finland, education is compulsory for all Finnish citizens until the age of 17 years. The conscripts entering the military service are relatively young, and most come straight from school or have only been in working life for a few years. Thus, occupational differences cannot fully explain the strong effect of education on back pain in this study. Another explanation for the strong correlation between education level and back pain is that educated people are more aware of healthy lifestyle habits and may consciously select them.^13^ Education also provide better problem-solving abilities and increase compliance with medical programmes.^12^

In this study, smoking was a clear risk factor for back pain and previous studies have also shown an association between smoking and back pain.^14^ Tobacco affects through atherosclerosis of the vessels that nourish the lumbar spine, which causes degenerative changes in the vertebrae and also directly affects cell proliferation and collagen fibrils locally.^15,16^ In addition to adults, smoking also increases the likelihood of developing back pain among adolescents.^17,18^ The first radiological signs of aortic atherosclerosis are apparent already in young adulthood, but the vascular alteration to the spine tends to build up later.^19^ For this reason, effective methods for preventing smoking may reduce the socioeconomical burden caused by back pain in addition to other health benefits.

In this study, BMI showed no correlation with back pain. Previous studies among Finnish conscripts and young people have shown a moderate correlation between high BMI and back pain.^20–22^ Vast majority of the conscripts were normal weight (BMI 25 or less), and only 7% of the conscripts were obese having BMI over 30. One explanation for the missing association between BMI and back pain in this study is that conscripts suffering from severe obesity are discharged from military service for health reasons. Data were not available of these discharged conscripts to verify this assumption. This study contained only young male adults, and the follow-up time was relatively short to reveal the impact of slowly varying variables, such as BMI, on back pain.

Back pain alone is a strong risk factor for future pack pain.^23,24^ In this study, previous back pain doubled the risk of future back pain and the same results have been found in a previous study.^24^ Unfortunately, the high prevalence of back pain makes prevention methods difficult.^24^

This study has many strengths. The Finnish military service is compulsory for all men and only 20% of conscripts either discontinue their service or choose civil service instead. This guaranteed a good generalized population sample of young Finnish adult males. The participants were randomly selected, and their medical records of all visits to the Garrison Health Care Centre were available. All the conscripts filled the same pre-service questionnaire, minimizing the possibility of selection bias through the questionnaire form. The sample size was also large.

A weakness of this study is that the service time changed during the study period, also causing alternation in the content and physical activities during service. The Finnish military service is only compulsory for men, and the number of women in the service is low. Because of the low number of female conscripts, they were excluded from this study. The coding method for diseases (ICD) also changed during the study period, and this might have had an impact on coding consistency. In addition, diagnoses were missing from 70% of the back-related visits, and these visits were recorded as back pain-related visits according to the reason for seeking care. Because of this, it was not possible to categorize the back pain visits into specific and non-specific back pain visits. Data were available only from Garrison Health Care of conscripts who completed the service. The lacking of data of conscripts discharged from the service may alter the BMI distribution and be reason for the correlation of back pain and BMI in this study. In the latest conscript group born in 1989, data collection and the data available for this cohort were limited, because nearly half of the conscripts had not yet started their service. On average, 80% of the men in this study participated in military service and the enrollment percentage is in line with Finnish long-term statistics.^5^

In conclusion, the percentage of conscripts with back-related problems in military service has remained stable at around 20% over the last 20 years. Smoking and musculoskeletal problems before military service tend to increase the prevalence of back pain among conscripts, and low education level and smoking are risk factors for developing back pain during service.

Therefore, smoking cessation and measures to increase education levels should be among the preventive methods aiming to decrease back pain incidence among young men.

## References

[1] Olafsson G , Jonsson E, Fritzell P, Hägg O, Borgström F: Cost of low back pain: results from a national register study in Sweden. Eur Spine J 2018; 27(11): 2875–81. 30155730 10.1007/s00586-018-5742-6

[2] Hestbaek L , Larsen K, Weidick F, Leboeuf-Yde C: Low back pain in military recruits in relation to social background and previous low back pain. A cross-sectional and prospective observational survey. BMC Musculoskelet Disord 2005; 26(5): 25. 10.1186/1471-2474-6-25 PMC1180830 15918894

[3] Mattila VM , Sahi T, Jormanainen V, Pihlajamäki H: Low back pain and its risk indicators: a survey of 7,040 Finnish male conscripts. Eur Spine J 2008; 17(1): 64–9. 17874146 10.1007/s00586-007-0493-9 PMC2365533

[4] Mattila V , Kuronen P, Pihlajamäki H: Nature and risk factors of injury hospitalization in young adults: a follow-up of 135,987 military conscripts. Scand J Public Health 2007; 35(4): 418–23. 17786806 10.1080/14034940601181439

[5] Tilastokeskus : Findikkaattori. 2017. Available at http://www.findikaattori.fi; accessed April 27, 2017.

[6] Frilander H , Miranda H, Mutanen P, Mertelin T, Pihlajamäki H, Viikari-Juntura E: Trends in musculoskeletal disorders and related health care utilization among conscripts in Finland, 1967-2006. Mil Med 2012; 177(9): 1069–74. 23025137 10.7205/milmed-d-11-00327

[7] Knox J , Orchowski J, Scher D, Owens B, Burks R, Belmont P: The incidence of low back pain in acite duty united states military service members. Spine 2011; 36(18): 1492–500. 21224777 10.1097/BRS.0b013e3181f40ddd

[8] Leino PI , Berg MA, Puska P: Is back pain increasing? Results from national surveys in Finland during 1978/9–1992. Scand J Rheumatol 1994; 23(5): 269–76. 7973482 10.3109/03009749409103728

[9] Taanila HP , Suni JH, Pihlajamäki HK, et al: Predictors of low back pain in physically active conscripts with special emphasis on muscular fitness. Spine J 2012; 12(9): 737–48. 22297262 10.1016/j.spinee.2012.01.006

[10] Latza U , Kohlmann T, Deck R, Raspe H: Influence of occupational factors on the relation between socioeconomic status and self-reported back pain in a population-based sample of german adults with back pain. Spine 2000; 1(25): 1390–7. 10.1097/00007632-200006010-00011 10828921

[11] Hoy D , Brooks P, Blyth F, Buchbinder R: The epidemiology of low back pain. Best Pract Res Clin Rheumatol 2010; 24(6): 769–81. 21665125 10.1016/j.berh.2010.10.002

[12] Suman A , Bostick GP, Schaafsma FG, Anema JR, Gross DP: Associations between measures of socio-economic status, beliefs about back pain, and exposure to a mass media campaign to improve back beliefs. BMC Public Health 2017; 504(17).doi: 10.1186/s12889-017-4387-4. PMC5445411 28545420

[13] Hagen KB , Holte HH, Tambs K, Bjerkedal T: Socioeconomic factors and disability retirement from back pain: a 1983–1993 population-based prospective study in Norway. Spine 2000; 25(19): 2480–7. 11013500 10.1097/00007632-200010010-00010

[14] Golberg MS , Scott SC, Mayo NE: A review of the association between cigarette smoking and the development of nonspecific back pain and related outcomes. Spine 2000; 25(8): 995–1014. 10767814 10.1097/00007632-200004150-00016

[15] Yanbaeva DG , Denteren MA, Creutzberg EC, Wesseling G, Wouters EF: Systemic effects of smoking. Chest 2007; 131(5): 1557–66. 17494805 10.1378/chest.06-2179

[16] Akmal M , Kesani A, Anand B, Singh A, Wiseman M, Goodship A: Effect of nicotine on spinal disc cells: a cellular mechanism for disc degeneration. Spine 2004; 29(5): 568–75. 15129075 10.1097/01.brs.0000101422.36419.d8

[17] Feldman DE , Rossignol M, Shrier I, Abenhaim L: Smoking: a risk factor for development of low back pain in adolescents. Spine 1999; 24(23): 2492–6. 10626312 10.1097/00007632-199912010-00011

[18] Feldman DE , Shrier I, Rossignol M, Abenhaim L: Risk factors for the development of low back pain in adolescence. Am J Epidemiol 2001; 154(1): 30–6. 11427402 10.1093/aje/154.1.30

[19] Kauppila LI : Atherosclerosis and disc degeneration/low-back pain- a systematic review. Eur J Vasc Endovasc Surg 2009; 37(6): 661–70. 19328027 10.1016/j.ejvs.2009.02.006

[20] Mikkonen PH , Jaana L, Remes J, Tammelin T: Association between overweight and low back pain. Spine 2013; 38(12): 1026–33. 23459137 10.1097/BRS.0b013e3182843ac8

[21] Taanila H , Suni J, Pihlajamäki H, Mattila V, Ohrankämmen O, Vuorinen P: Aetiology and risk factors of musculoskeletal disorders in physically active conscripts: a follow-up study in Finnish Defence Forses. BMI Musculoskelet Disord 2010; 5(11): 146. 10.1186/1471-2474-11-146 PMC2911403 20602765

[22] Frilander H , Solovieva S, Mutanen P, Pihlajamäki H, Heliövaara M, Viikari-Juntura E: Role of overweight and obesity in low back disorders among men: a longitudinal study with a life course approach. BMJ Open 2015; 5(8).doi: 10.1136/bmjopen-2015-007805 PMC4550727 26297359

[23] Rossignol M , Lortie M, Ledoux E: Comparison of spinal health indicators in predicting spinal status in a 1-year longitudinal study. Spine 1993; 18(1): 54–60. 8434325 10.1097/00007632-199301000-00009

[24] Papageorgiou AC , Croft PR, Thomas E, Ferry S, Jayson M, Silman AJ: Influence of previous pain experience on the episode incidence of low back pain: results from the South Manchester Back Pain Study. Pain 1996; 66(2-3): 181–5. 8880839 10.1016/0304-3959(96)03022-9

